# Molecular characterization of African swine fever viruses from Burkina Faso, 2018

**DOI:** 10.1186/s12917-022-03166-y

**Published:** 2022-02-12

**Authors:** Moctar Sidi, Habibata Lamouni Zerbo, Bruno Lalidia Ouoba, Tirumala Bharani K. Settypalli, Gregorie Bazimo, Hamidou Sandaogo Ouandaogo, Boubacar N’paton Sie, Ilboudo Sidwatta Guy, Drabo Dji-tombo Adama, Joseph Savadogo, Anne Kabore-Ouedraogo, Marietou Guitti Kindo, Jenna E. Achenbach, Giovanni Cattoli, Charles E. Lamien

**Affiliations:** 1 Laboratoire National d’Elevage, Ouagadougou, Burkina Faso; 2 Animal Production and Health Laboratory, Joint FAO/IAEA Division of Nuclear Techniques in Food and Agriculture, Department of Nuclear Sciences and Applications, International Atomic Energy Agency, Vienna, Austria; 3 Direction Générale Des Services Vétérinaire, Ouagadougou, Burkina Faso; 4 Battelle Memorial Institute, 1001 Research Park blvd, Charlottesville, VA 22901 USA

**Keywords:** African swine fever, Central variable region, B646L, B602L, E183L, Burkina Faso

## Abstract

**Background:**

African swine fever (ASF) is a viral hemorrhagic disease of domestic and wild swine. ASF has been endemic in Burkina Faso since 2003. In October 2018, substantial pig deaths occurred in Ouagadougou and two neighboring municipalities in central Burkina Faso. Following these mortalities, the veterinary extension services carried out investigations to begin control measures and collect samples.

**Methods:**

We performed real-time PCR for diagnostic confirmation and molecular characterization of the virus based on the partial P72, the complete p54, the partial CD2v, and partial B602L genes.

**Results:**

The field study revealed that mortalities started two weeks before our investigations. The real-time PCR results confirmed ASFV DNA in twenty samples out of sixty-two blood samples collected in four different locations. The sequencing and phylogenetic analysis showed that ASFVs causing these outbreaks belong to genotype I and serogroup 4. The study of the CVR showed 4 TRS variants, and that of the CD2v amino acid sequence revealed five variants based on the number of deleted KCPPPK motifs in the C-terminal proline-reach region of the protein.

**Conclusions:**

The existence of multiple variants in these outbreaks shows the importance of molecular characterization to understand the evolution of ASFV isolates and the link between epidemics.

## Background

African swine fever (ASF) is a viral disease caused by the ASF virus (ASFV), an Asfivirus of the family Asfarviridae and can affect both wild and domestic pigs.

ASF causes a drop in animal production and affects livestock productivity due to high mortality that can reach 100% in outbreaks involving highly virulent strains. Due to this high mortality, the disease has a significant impact on low-income countries.

Globally, ASF continues to spread and has expanded its geographical distribution to four continents: Africa, Europe, Asia, and America [1, 2].

In West Africa, ASF was first reported in 1978 in Senegal and spread to Ivory Coast in 1996, followed by Cape Verde, Togo, and Nigeria in 1997, Benin and Ghana in 1999, and Burkina Faso in 2003 [3–7]. In 2018 alone, Benin, Ivory Coast, Niger, Nigeria, Togo, Ghana, Senegal, Bissau Guinea reported 79 outbreaks [8].

Burkina Faso has an estimated pig population of 2,345,803 [9]. ASF is now endemic in the country, affecting the livelihood of smallholder farmers and food security at a national level. For instance, Burkina Faso reported 32 ASF outbreaks to OIE between 2014 and 2018, affecting 14 provinces.

Since there is no vaccine for ASF, disease control mainly relies on early detection and implementation of control measures such as animal movement restriction, restricted access to infected areas, stamping out, and proper carcass disposal [10]. Besides, is equally important to consistently characterize outbreak strains to assess how the disease is spreading to better implement control measures. There are molecular tools capable of detecting all ASFV genotypes, serogroups, and additional gene variant analysis to characterize strains within various genotypes [11–15].

Currently, there are twenty-four known ASFV genotypes [16, 17], all of which have been detected in Africa. Europe and Asia [18] has experienced both genotypes I and II.

**Fig.1:**
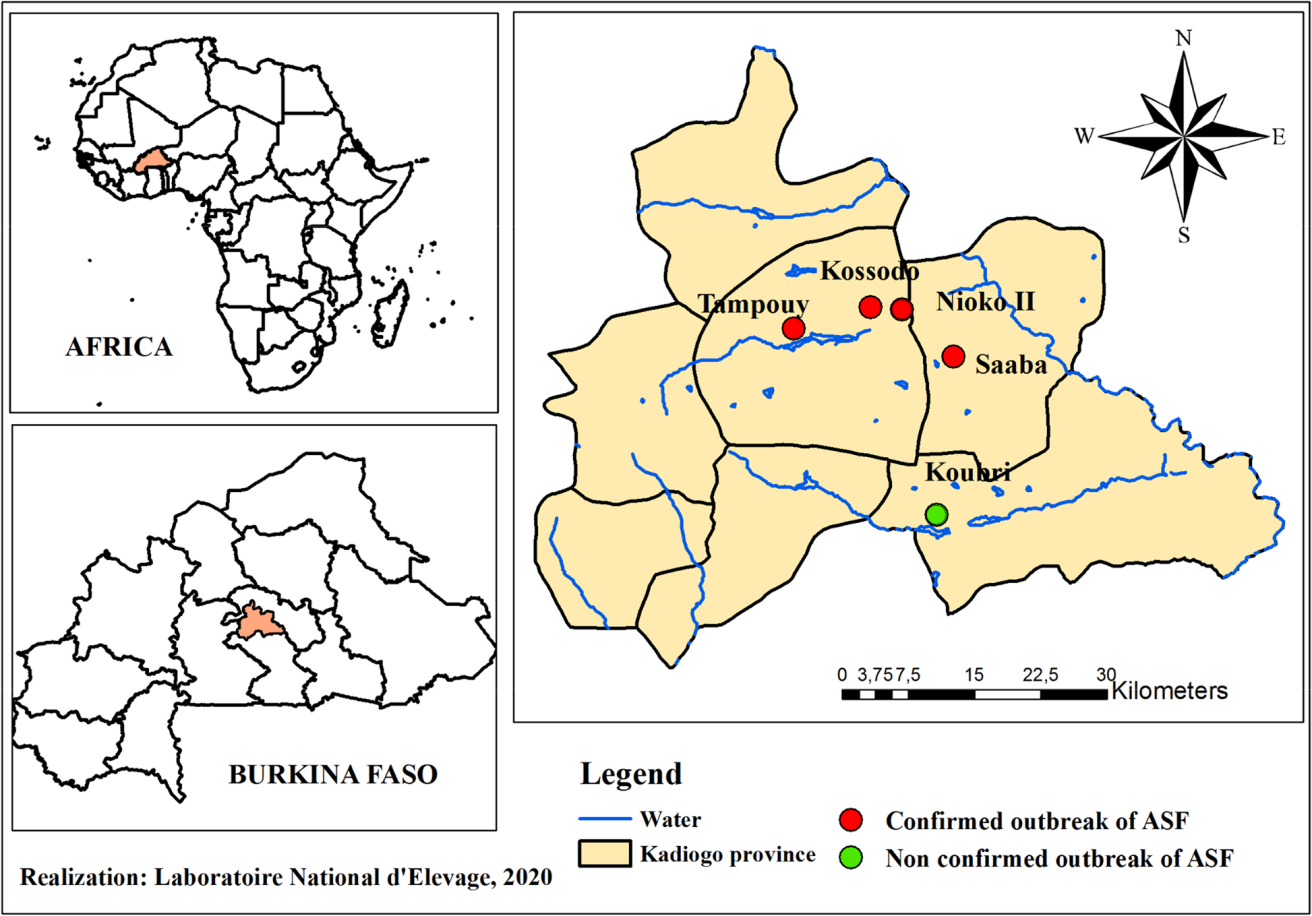
Map showing the ASF outbreak suspicion areas in Ouagadougou and surroundings (Kadiogo province) in central Burkina Faso. The red zones circle indicates the confirmed outbreaks, and the green the non-confirmed ones

In October 2018, there was a report of high mortality of pigs in Ouagadougou and surrounding areas in the Kadiogo province (Fig. 1), prompting the deployment of field investigation teams from the veterinary extension services to implement control measures.

The present study details our findings during the field investigation and the molecular characterization of isolates collected during these outbreaks.

## Materials and Methods

### Study area, outbreak investigation, and sample collection

The study covered four farms in the peri-urban region of Ouagadougou (Fig. 1). All suspicions of ASF cases were from semi-intensive farms with a herd size of 30 to 100 pigs. The field investigations collected information on farm management, history of diseases, examination of dead and sick animals, and sample collection. Blood samples were collected on EDTA-treated tubes from Sixty-two sick animals at different locations in Ouagadougou (Table 1) and taken to the Laboratoire National d'Elevage, Ouagadougou, for diagnostic confirmation.

**Table 1:** Outbreak informations and samples of this study

Sampling location	Morbidity	Mortality	Sample collect date	Number of samples collected
Ouagadougou/ Kossodo	100%	91%	10 October 2018	10
Ouagadougou/ Nioko II	100%	100%	10 October 2018	15
Saaba	30%	100%	02 October 2018	16
Ouagadougou / Tampouy	100%	57%	18 October 2018	12
Koubri				9
**Total**				**62**

### Nucleic acids extraction and amplification

DNA was extracted from blood using DNeasy® Blood & Tissue kit (Qiagen, Hilden, Germany), according to the manufacturers' instructions. ASFV DNA detection was performed by real time PCR, using an adaptation of a previously described procedure [19].

### Molecular characterization of African swine fever virus

Three different genomic targets of the ASFV genome were amplified and sequenced using previously described methods with primers: P72-U- 5`-GGCACAAGTTCGGACATGT-3` and P72-D—5`-GTACTGTAACGCAGCACAG-3` [20] for the C-terminal region of the B646L gene encoding the p72 protein, P54F-5'-GCCTGCGGATTCTGAAGATA-3'and P54R- 5`-AGGACGCAATTGCTTAAACG -3` [12] for the complete E183L gene encoding the p54 protein, ORF9RLW_F-5`-AATGCGCTCAGGATCTGTTAAATCGG-3` and ORF9RLW_R—5`-TCTTCATGCTCAAAGTGCGTATACCT -3` [21] for the central variable region (CVR) of the B602L gene.

To determine the serogroups of the 2018 ASFV isolates from Burkina Faso, the partial CD2v gene [22] was amplified and sequenced using two sets of primers ga3611for- 5`-TATAATATAACAAATAATTGTAG-3`, ga4220rev-5`-AGGGACGCATGTAGTAAATAG-3`, ga4124f-5`-CTGAATCTAATGAAGAAGA-3` and ga4698r-5`-AAGTCTTTGTAGGTTTTTCGTTCA-3` to generate two overlapping fragments. Briefly, a mixture consisting of 12. 5 µL of the 2X Q5 High-Fidelity Master Mix (Neb Inc), 500 nM of each primer, and 2 µL of the template DNA was set up in a total reaction volume of 25 µL. The following thermal profile was used for amplification: initial denaturation at 95 °C for 5 min, then 40 cycles of denaturation at 95 °C for 45 s, annealing at 52 °C for 45 s and elongation at 72 °C for 90 s, and final elongation at 72 °C for 5 min.

The amplified PCR products were purified using Wizard SV Gel and PCR Clean-Up kit, according to the manufacturer's protocol (Promega Corporation, Madison, WI, USA) and sequenced by LGC Genomics (Berlin, Germany). The raw sequences were assembled and edited using Vector NTI 11.5 Software (Life Technologies, Carlsbad, CA, USA). The nucleotide sequences of ASFV isolates of Burkina Faso were deposited in GenBank under accession numbers MT851949 to MT851967 (p54 gene), MT851968 to MT851986 (p72 gene), MT851987 to MT852005 (B602L gene), and MT852006 to MT852019 (CD2v gene). For comparative analysis, additional ASFV sequences were retrieved from GenBank. Multiple sequence alignments were performed with MUSCLE as implemented in MEGA software version 7 [23].

For phylogenetic analysis of the p72 gene fragments, a data set of 93 nucleotide sequences (398 characters) was prepared, including 19 sequences from this study and additional sequences from GenBank with at least one representative of each of the 24 known ASFV genotypes and the sequences of some historical samples from Burkina Faso and neighboring countries. A Neighbor-Joining (NJ) tree was produced for the p72 gene in Mega 7 using the Maximum Composite Likelihood method, with the data being re-sampled 1,000 times using the bootstrap method.

For the p54 tree, the dataset consisted of 63 taxa (453 characters), including 19 sequences from this study and additional sequences from GenBank with at least one representative of each of the 18 ASFV p72 genotypes for which a p54 sequence is available. Historical sequences from Burkina Faso and neighboring countries were also included in the dataset. A Minimum Evolution tree was constructed using the p-distance substitution model and the Close-Neighbor-Interchange (CNI) algorithm. The initial tree was generated using the Neighbor-joining algorithm. All positions with less than 95% site coverage were removed. The data were re-sampled 1,000 times using the bootstrap method.

A maximum‐likelihood (ML) tree of the partial CD2v amino acid sequences was constructed applying the predetermined CpREV + G model. The dataset included representatives of the eight known ASFV serogroups and ASFVs clustering outside the eight established serogroups. For each phylogenetic reconstruction, the robustness of the tree topology was assessed using 1000 bootstrap replicates.

For each isolate, the CVR nucleotide sequence was translated into amino acid, and the deduced amino acid tetramers were matched with previously reported codes [11, 13, 24–26]. The CVR sequences were analyzed together with those of historical isolates from Burkina Faso and other Western African countries.

## Results

### Outbreak investigations

The affected farms consisted of semi-intensive production systems with open-air housing and free-ranging animals kept under reduced biosecurity levels. The investigations revealed that the disease had started several days to two weeks prior to the visit by our field team to the Saaba farm. The affected farms had introduced new animals without observing an initial quarantine period. In addition, several butchers visited the farms to acquire animals for slaughtering. Typically, the butchers visit several farms to obtain pigs, then transport the purchased animals to meat processing sites increasing the risk for further disseminating the disease. In three farms located in the peri-urban area of Ouagadougou, 100% morbidity was observed, with a fatality rate varying between 57 and 100% (Table 1). The farm in Saaba had low morbidity, however, it presented 100% mortality (Table 1). The first outbreak occurred in Saaba, followed by Nioko II, Kossodo, and Tampouy. These four localities have in common a network of roads connecting them, thus facilitating the trade of animals between the sites and connecting them to pig slaughtering sites. The diseased pigs showed depression, inappetence, and skin redness, especially on the ears with petechiae. They also had hyperthermia, reduced mobility, and lethargy. Unfortunately, fresh carcasses were not available for necropsy.

### Laboratory diagnosis

The real-time PCR results confirmed ASFV DNA in twenty, out of sixty-two blood samples, from the four different locations (Fig. 1) and distributed as follows: seven positives in Kossodo, seven in Nioko II, three in Saaba, and three in Tampouy. Nine samples from Koubri tested negatives.

### Molecular characterization

We successfully sequenced nineteen partial p72 and p54 genes, fourteen partial CD2v genes, and twenty partial B602L.

**Fig. 2:**
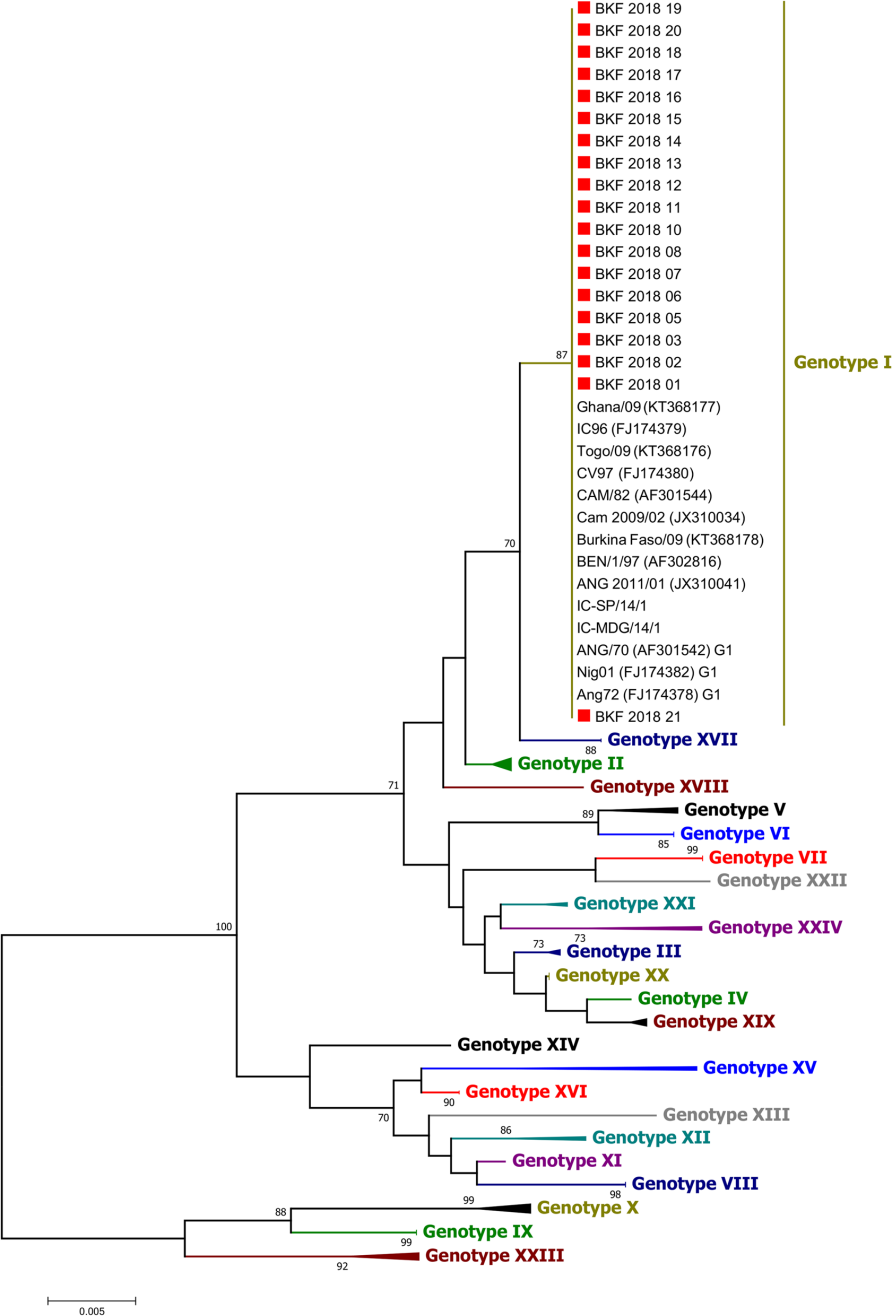
Neighbor-joining tree of the partial *p72* gene, depicting genetic relationships of the 2018 ASFV isolates from Burkina Faso (highlighted with red diamonds) with representatives of the 24 known ASFV genotypes. The evolutionary distances were computed using the Maximum Composite Likelihood method. Bootstrap values > 70% are shown

**Fig. 3:**
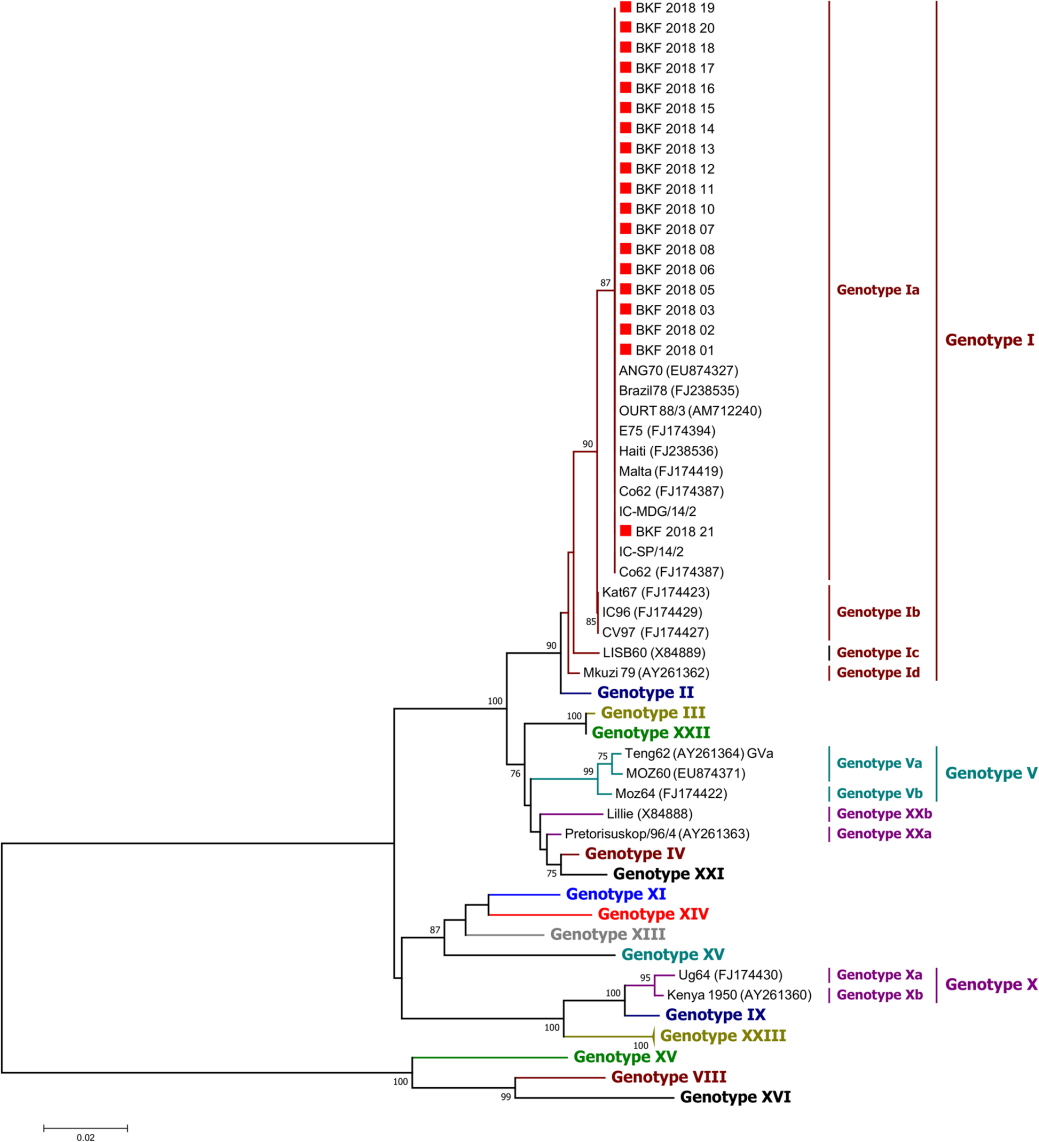
Minimum Evolution tree, based on the full-length p54 gene sequences, depicting the genetic relationships between the 2018 ASFV isolates from Burkina Faso (are highlighted with red diamonds) with representatives of the 18 out of 24 known ASFV genotypes. The evolutionary distances were computed using the p‐distance method. Bootstrap values > 70% are shown

**Fig. 4:**
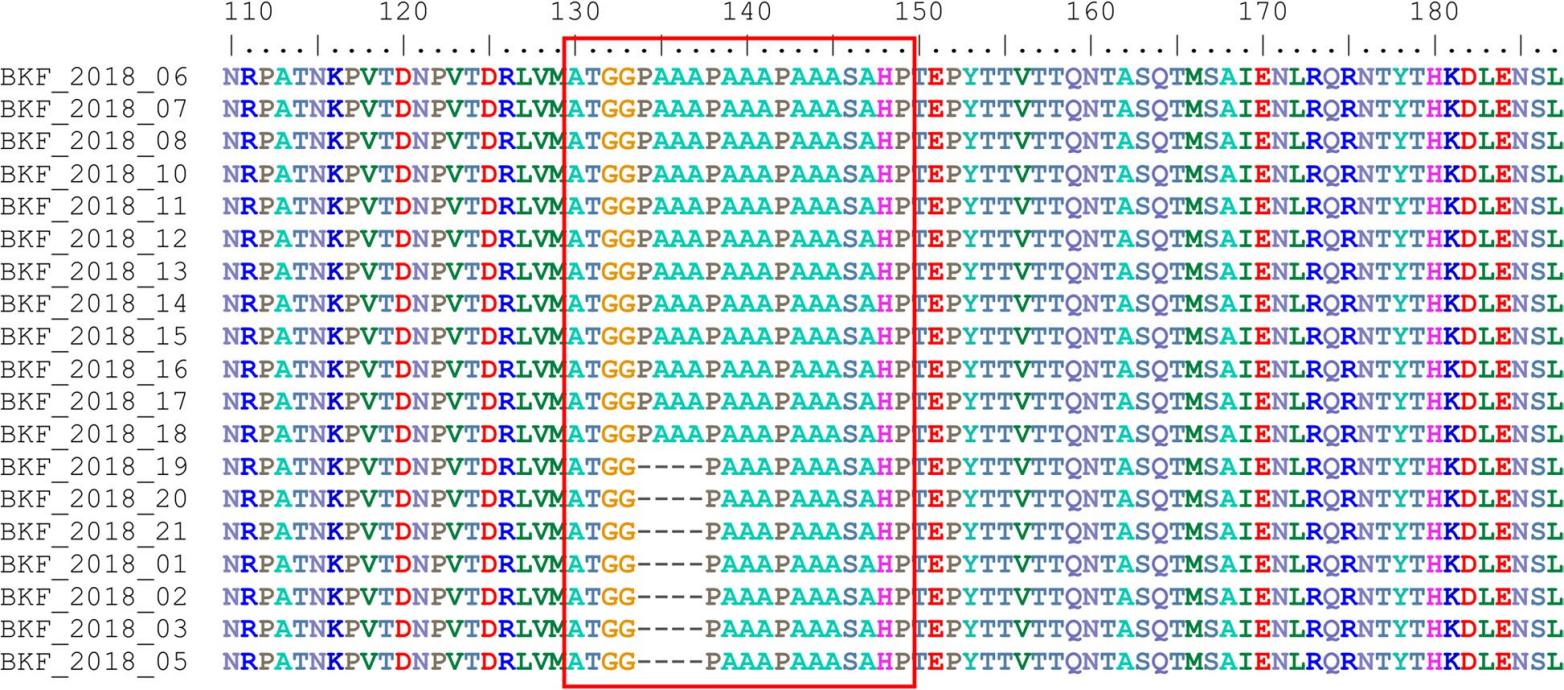
Comparison of the 2018 ASFV isolates from Burkina Faso using the amino acid sequences of the p54 protein. The partial representation of the multiple sequence alignment shows the amino acid sequences variation among the isolates (inside the red box)

In the p72 phylogenetic analysis, all 2018 ASFV from Burkina Faso clustered within ASFV genotype I (Fig. 2), suggesting that only genotype I ASFVs caused these outbreaks. The analysis of the p54 sequences showed that all Burkina Faso ASFV isolates of this study belong to the genotype Ia subgroup (Fig. 3). However, a close inspection of the multiple sequences alignments of the nucleotide and amino acid sequences of the P54 gene showed that, although they all belong to genotype Ia, the 2018 isolates of Burkina Faso comprised two sub-groups based on the complete length of the p54 gene (552 bp, and 564 bp). The difference was due to a deletion of 12 nucleotides. The amino acid sequences showed that the deletion occurred in a region flanked by "ATGG" and "SAHP" and containing a series of 2 or 3 repeats of the four amino acids "PAAA" (Table 2) and Fig. 4.

**Table 2:** Comparison of the CVR, p54 and CD2v (C-terminal) profiles of the 2018 ASF outbreak isolates from Burkina

Isolate name	CVR	Number of repeats (CVR)	p54 (PAAA repeats)	CD2v (number of deleted PCPPPK)	Location	Outbreak date
BKF_2018_01	ABNAAAAAAACAAAAAACBNAFA	23	3	2	Kossodo	28-Sep-18
BKF_2018_02	ABNAAAAAAACAAAAAACBNAFA	23	3	2	Kossodo	28-Sep-18
BKF_2018_03	ABNAAAAAAACAAAAAACBNAFA	23	3	2	Kossodo	28-Sep-18
BKF_2018_05	ABNAAAAACBNAAAAACBNAAAAAAACBNAFA	32	3	4	Kossodo	28-Sep-18
BKF_2018_06	ABNAAACBNAFA	12	2	0	Kossodo	28-Sep-18
BKF_2018_07	ABNAAACBNAFA	12	2	0	Kossodo	28-Sep-18
BKF_2018_08	ABNAAACBNAFA	12	2	0	Kossodo	28-Sep-18
BKF_2018_09	AAAABNABBNABBAABBNABNABA	24	2	ND	Nioko II	29-Sep-18
BKF_2018_10	ABNAAACBNAFA	12	2	0	Nioko II	29-Sep-18
BKF_2018_11	ABNAAACBNAFA	12	2	0	Nioko II	29-Sep-18
BKF_2018_12	ABNAAACBNAFA	12	2	0	Nioko II	29-Sep-18
BKF_2018_13	ABNAAACBNAFA	12	2	0	Nioko II	29-Sep-18
BKF_2018_14	ABNAAACBNAFA	12	2	0	Nioko II	29-Sep-18
BKF_2018_15	ABNAAACBNAFA	12	2	0	Nioko II	29-Sep-18
BKF_2018_16	ABNAAACBNAFA	12	2	3	Saaba	23-Sep-18
BKF_2018_17	ABNAAACBNAFA	12	2	3	Saaba	23-Sep-18
BKF_2018_18	ABNAAACBNAFA	12	2	3	Saaba	23-Sep-18
BKF_2018_19	ABNAAAAACBNAAAAACBNAAAAAAACBNAFA	32	3	4	Tampouy	11-Oct-18
BKF_2018_20	ABNAAAAACBNAAAAACBNAAAAAAACBNAFA	32	3	4	Tampouy	11-Oct-18
BKF_2018_21	ABNAAAAACBNAAAAACBNAAAAAAACBNAFA	32	3	4	Tampouy	11-Oct-18

**Fig. 5:**
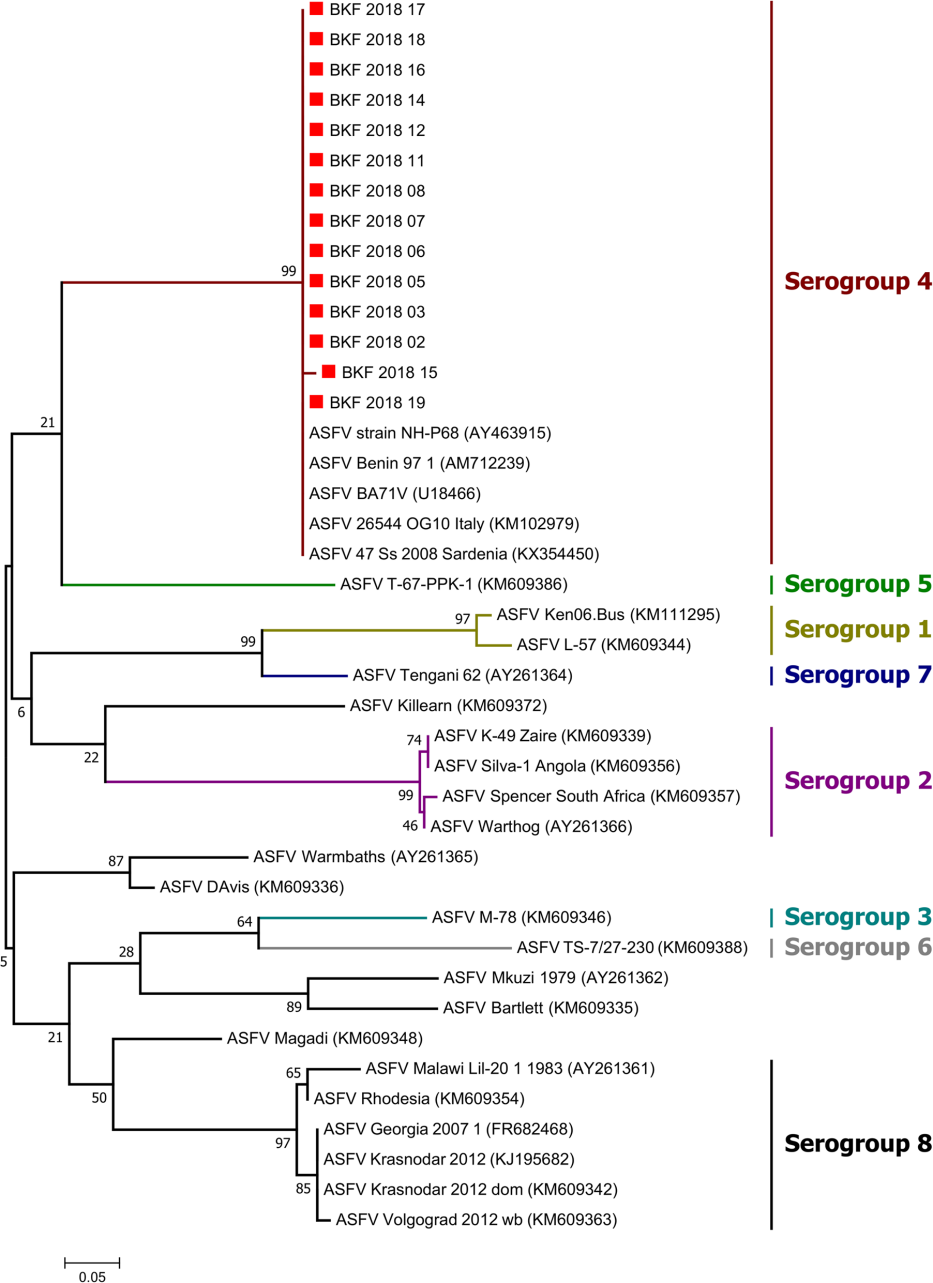
Maximum Likelihood tree based on the partial amino acid sequences of the CD2v protein. The tree shows the relationship between the 2018 ASFV isolates from Burkina Faso (highlighted with red diamonds) and the representatives of the eight known ASFV serogroups and ASFVs clustering outside the eight established serogroups. The General Reversible Chloroplast Model with Gamma distribution (cpREV + G) was used. Bootstrap values higher than 70% are shown

**Fig. 6:**
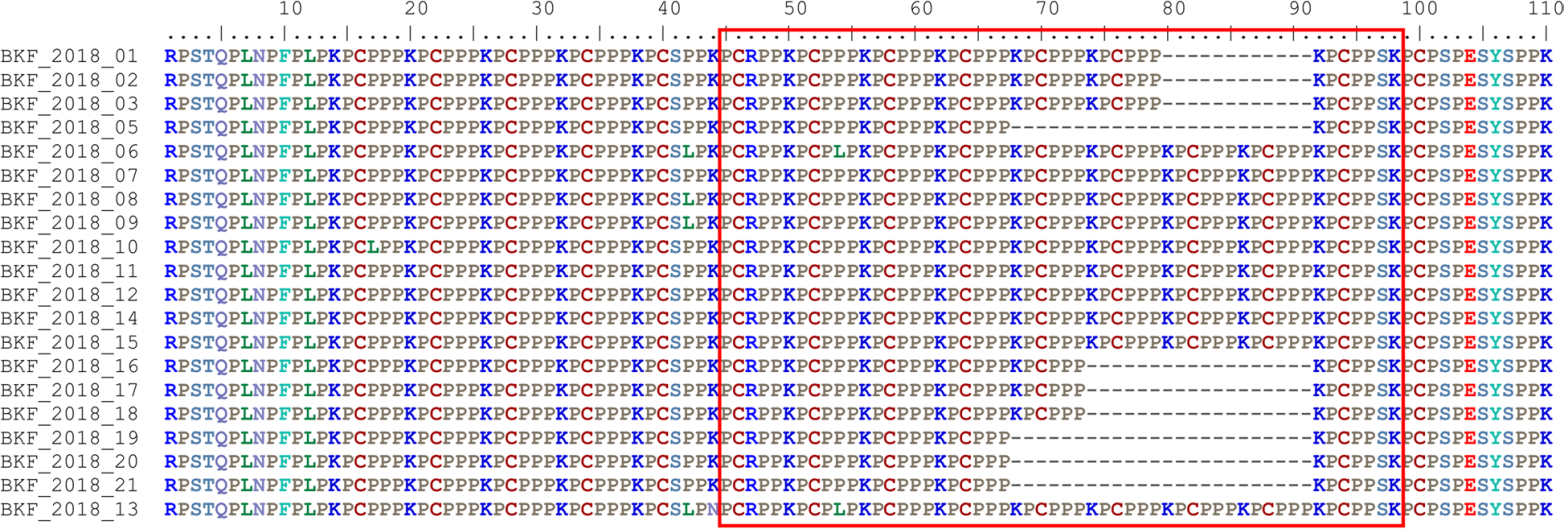
Comparison of the 2018 ASFV isolates from Burkina Faso using the C-terminal of the CD2v protein. The partial representation of the multiple sequence alignment shows the amino acid sequences variation in the C-terminal of the CD2v protein (inside the red box)

The phylogenetic tree of the CD2v partial amino acid sequences showed that all sequenced isolates belong to serogroup 4 (Fig. 5). The multiple sequence alignment of the amino acid sequences of the CD2v protein showed that the proline-rich region near the C-terminal comprised variable number repeated units of KPCPPP (Fig. 6). Therefore, based on the number of deleted PCPPPK units, the isolates could be segregated into groups 0, 2, 3, or 4 missing units of the KPCPPP repeats (Table 2). Three variants with 0, 2, and 4 missing KPCPPP units were present on the farm at Kossodo. The ASFVs in Saaba had three missing KPCPPP units, those in Tampouy had four missing KPCPPP units, and those in Nioko II had 0 units missing.

The analysis of 20 CVRs revealed four variants of the tetrameric repeat sequence (TRS) with 32, 24, 23, and 12 TRS (Table 2).

Three out of the four CVR variants of this study (Table 2) shared motifs beginning with "ABNAAA" and ending with "CBNAFA" flanking additional TRS. The CVR with 12 TRS was present in Saaba, the index outbreak (September 28, 2018), and in two subsequent outbreaks in Nioko II and Kossodo (Table 2). In Nioko II, there was an additional profile with 24 TRS, unique to this farm, for which, unfortunately, we could not sequence the p72. In Kossodo, there were two other profiles with 23 and 32 TRS, the former being identical to the pattern found in the fourth outbreak that occurred on October 11, 2018, in Tampouy.

## Discussion

Since its first discovery in 2003 [5], ASF has been endemic in Burkina Faso.

An epizootic of African swine fever in 2008, and the one in 2018, led to significant losses in the pig production sector of the country and neighboring countries of Benin, Ivory Coast, Niger, Togo, and Ghana.

The trade of live animal and animal products and intensive movement of people between the porous borders of these countries can explain the transboundary persistence of ASF.

Our finding that ASF genotype I caused these outbreaks is consistent with previous reports showing that this genotype was the only one circulating in West Africa [16, 27–29].

Nonetheless, it is worth noting that ASFV genotype II has been reported recently in Nigeria [30]. In central Africa, only genotype I ASFV is present in Cameroon [31]. Other Central African Countries also have genotype I, co-circulating with genotype IX in Chad and the Central African Republic, and genotype IX and XIV in DRC [12]. The analysis of the CVR in the 2018 samples showed four variants of the tetrameric repeats. An earlier study of samples collected between 2007 and 2010 [28] showed ten variants of the tetrameric repeats, all different from those found in this study, making fourteen variants identified in the country, between 2007 and 2010 and 2018.

In this study, there were three TRSs variants, two p54 profiles, three CD2v variants in Kossodo, and two different TRSs variant Nioko II.

Saaba, Nioko II, and Kossodo shared a common ASFV variant with 12 TRS in the CVR, suggesting an epidemiological link between those three outbreaks. The variant with 12 TRS from Saaba had three KPCPPP units deleted at the C-terminal of its CD2v, differentiating it from the 12 TRS variants of Nioko II and Kossodo with no missing KPCPPP unit. Saaba, Nioko II, and Kossodo are three nearby locations with direct connections and frequent trade activities. The onset dates of these outbreaks, between September 23 and September 28, 2018, support the hypothesis that the disease spread from the first outbreak in Saaba to the neighboring sites of Nioko II and Kossodo. Similarly, ASFV BKF_2018_05, a 32 TRS variant collected in Kossodo on September 28, 2018, had identical TRS, CD2v, and p54 profiles to all ASFV collected in Tampouy during an outbreak that started on October 11, 2018. The detection of identical viruses and the date of the outbreaks in the two locations suggest a spread of ASFV from Kossodo to Tampouy.

It is unclear why multiple ASFV variants are present in Kossodo and Nioko II but could suggest multiple introductions of the disease within those farms. It is also possible that the virus mutated while spreading within those farms. Previous reports have suggested changes in the CVR sequence during adaptation of ASFV to cell cultures [21] and during ASF epidemics [12, 16].

Our study suggests that the proline-rich region near the C-terminal of the CD2v gene is suitable for genotype I ASFV isolates discrimination.

Using genotype I and serogroup 4 isolates recovered during 2018 outbreaks in central Burkina Faso, we have shown that the number of deleted KPCPPP units in the proline-rich region of the CD2v protein varied substantially, enabling it to be an additional mean to discriminate ASFVs. Hence, our study confirms an earlier report that analysis of the number of PCPPPK repeats provided an additional mean, similar to the analysis of the CVR profile [32]. Further investigations will establish whether this approach is suitable to analyses of other ASFV serogroups and genotypes.

Surprisingly, our field investigations determined that the pig breeders know little about common diseases of swine, especially African swine fever. In addition, there is insufficient technical support and minimal effort to increase pig breeder and community awareness of ASF.

The pig production system in Burkina Faso is based mainly on a traditional extensive system with household free-ranging pigs and small to medium semi-intensive farming systems mainly in peri-urban areas. The housing comprises shelters made with local material in extensive systems or semi-modern to modern housing in the semi-intensive system [33]. In most farms, brewers' grains and swill are the primary feeds for the pigs.

Near and between the farms, there is a lack of proper biosecurity practices, promoting several diseases, including ASF [33]. Additionally, other domestic animals like cattle, sheep, goats, dogs, rodents, and birds are free-ranging and share common space with pigs; therefore, they could also spread diseases. These conditions raise the need for increased awareness of transboundary animal diseases and continued rapid diagnostics and analysis to identify and understand future outbreaks. For instance, additional outbreaks of ASF occurred in new locations in the country since those described in this paper. Although there was no evidence for an epidemiological link between the recent outbreaks and those described in this paper, the continuous occurrence of ASF outbreaks highlights inadequate disease management. This instigated the veterinary authorities to start implementing active surveillance to better monitor ASF and characterize the circulating genotypes of the ASF virus.”

## Conclusion

We have shown that ASFVs belonging to genotype I, serogroup 4, caused four outbreaks in October 2018 in Burkina Faso. There were 4 TRS variants based on the CVR analysis and five based on the number of deleted KCPPPK motifs in the C-terminal proline-reach region of the CD2v protein, showing that the latter can serve as an additional tool for ASFV isolates discrimination. Further studies are needed to understand how these variants have emerged. The presence of multiple variants involved in these outbreaks shows the importance of molecular characterization to understand the evolution of ASFV isolates and the link between epidemics.

## Data Availability

The data that support the findings of this study are openly available in NCBI at https://www.ncbi.nlm.nih.gov/nucrore

## Abbreviations

ASF: African swine fever
ASFV: African swine fever virus
CVR: Central Variable Region
CNI: Close-Neighbor-Interchange
DNA: Deoxyribonucleic acid
DRC: Democratic Republique of Congo
NJ: Neighbor-Joining
TRS: Tetrameric Repeat Sequence
